# Genome-wide association study for resistance to *Macrophomina phaseolina* in maize (*Zea mays* L.)

**DOI:** 10.1038/s41598-025-87798-8

**Published:** 2025-03-06

**Authors:** Gizem Oder, Semiha Yuceer, Canan Can, Muhammed Bahattin Tanyolac, Duygu Ates

**Affiliations:** 1 Department of Bioengineering, Ege University, Izmir, Turkey; 2 Phytopathology Department, Biological Control Research Institute, Adana, Turkey; 3 Department of Biology, Gaziantep University, Gaziantep, Turkey

**Keywords:** *Zea mays* L., GWAS, Maize, *Macrophomina phaseolina*, Resistance, SNP, Genetics, Agricultural genetics

## Abstract

Maize (*Zea mays* L.) is a frequently used food source in human and animal nutrition. *Macrophomina phaseolina* is a fungal pathogen causing charcoal rot disease in many plants, especially maize. This pathogen causes high yield losses in maize. The development of resistant maize genotypes is of great importance in controlling this disease. In this study, the population structure of 120 different maize genotypes with varying levels of disease resistance was determined and genome-wide association studies were performed. Each genotype was subjected to the pathogen under controlled conditions and their phenotypic responses to the disease were analyzed. Afterwards, single nucleotide polymorphisms were determined by DArT-seq sequencing. After filtering the SNP data, 37,470 clean SNPs were obtained. The population structure was analyzed with STRUCTURE software, and it was determined that the population was divided into two subgroups. The relationship between phenotypic and genotypic data was analyzed using the MLM (Q + K) model in TASSEL software. As a result, seven SNPs markers located on four different chromosomes were associated with disease resistance. The related markers can be used in the future for the development of maize varieties resistant to *M. phaseolina* by marker-assisted selection.

**Supplementary Information:**

The online version contains supplementary material available at 10.1038/s41598-025-87798-8.

## Introduction

Maize (*Zea mays* L. or corn) is a cross-pollinated, annual, and diploid (2n = 20) member of Poaceae family^1^ and has an average genome size of 2.4 Gb^2^. Maize is frequently used in human and animal nutrition due to its high content of vitamins A, B, and C, carbohydrates, protein, and phytochemicals^3,4^. In 2022, maize ranks as the second most produced cereal crop, covering a cultivation area of 43 million hectares globally. It is cultivated in 164 countries, where the annual harvest comes to the amount of about 1.16 billion tons^5^. Türkiye is one of the world’s important maize producers with approximately one million hectares and a production of 8.5 million tons annually. Considering the size of maize production worldwide, yield losses can have substantial economic consequences. Nevertheless, several factors negatively affect maize production with some of the major factors being diseases and pests.

Plant diseases caused by fungal infections are the most important pathogenic agents. *Macrophomina phaseolina* (*Mp*) is a broad-host fungi that can infect hundreds of different plants and induce a root cancer called charcoal rot in its host^6^. This pathogen is one of the most destructive diseases that can cause severe yield losses in maize plants and lead to 25–33% or more crop losses. Besides causing plant mortality, it adversely impacts the quality attributes of the product. A recent study observed that high disease severity caused by *Mp* caused a 30–70% decrease in the biophysical and biochemical properties of plants^7^. This highlights the significant adverse economic impact of the pathogen on plants, primarily due to reductions in both yield and quality. *Mp* infection can cause mortality both before and after plant emergence. The most prominent symptom is ‘blackleg’ due to sclerotia at the plant crown. Infected plants are characterized by lesions with dark margins and light grey centres^6^, wilted appearance and discoloured leaves^8^. Furthermore, the phytotoxic metabolites released by *Mp* cause vascular obstructions that impede nutrient transport, leading to plant death. The ability of this pathogen to persist in soil for long periods and its wide genetic diversity contributes to its widespread in different climatic conditions^9^.

The development of resistant cultivars to control *Mp* has become a critical requirement due to the limitations of current control methods. Chemical control can lead to resistance development of the pathogen and environmental problems; in addition, the effectiveness of systemic fungicides is limited due to their inability to reach the roots. Physical control methods require high cost and labour, which negatively affects agricultural productivity^10^. Although agronomic practices aim to reduce the density of microsclerotia, some methods such as irrigation are not effective^11,12^. Biological control varies depending on environmental conditions and does not always give reliable results. Although plant defense stimulants trigger defense mechanisms, their effects vary depending on genotype and environmental conditions, and in some cases may negatively affect other plant traits^13^. Consequently, due to the limitations of current methods, the development of resistant cultivars appears to be the most effective and sustainable strategy for permanent control of *Mp*.

Conventional breeding methods require many years to develop disease-resistant varieties in plants. However, modern molecular techniques such as genome-wide association studies (GWAS) accelerate this process and make significant contributions to breeding studies. GWAS reveals the relationship between genetic variants and plant traits using genotypic and phenotypic data. Since this technique directly identifies the genetic factors affecting phenotypic variation, it provides more precise and efficient results compared to traditional methods^14^. In particular, in previous studies, diseases such as Fusarium-induced ear rot^15^, *Aspergillus flavus* infection^16^, *Phytium aristosporum*-induced stalk rot^17^ and maize rough dwarfism^18^ were investigated by GWAS and resistance genes were identified. In addition, GWAS has also been used in the development of resistant varieties against the western corn rootworm^19^. Thus, it enables a better understanding the genetic background of complex traits such as disease resistance. By measuring the contribution of specific genetic variants to a trait, GWAS accelerates the identification of genetic markers to be used in breeding programs. This allows the development of resistant varieties in a shorter time.

One of the technologies used to provide genotypic data for GWAS is Diversity Array Technology (DArT). DArT detects thousands of single nucleotide polymorphism (SNP) markers quickly and efficiently without the need for whole genome sequencing^20^. SNP markers produced by DArT are used for mapping gene loci that control phenotypic traits and for the identification of genetic variants. Therefore, DArT and similar technologies make breeding programs more effective by providing genetic data to GWAS studies.

This study aimed to perform GWAS analysis to identify molecular markers for *Mp* resistance using 120 different maize genotypes. The research aims to identify genetic variations contributing to *Mp* resistance among different genotypes and specific SNPs associated with these variations. The identification of these molecular markers will facilitate future studies by providing an understanding of the genetic basis of *Mp* resistance. Furthermore, the findings will contribute to the development of breeding programs for the development of resistant varieties, ultimately strengthening plant protection strategies against *Mp* infection and contributing to sustainable agricultural practices.

## Results

### Phenotypic variations of the population

**Fig. 1:**
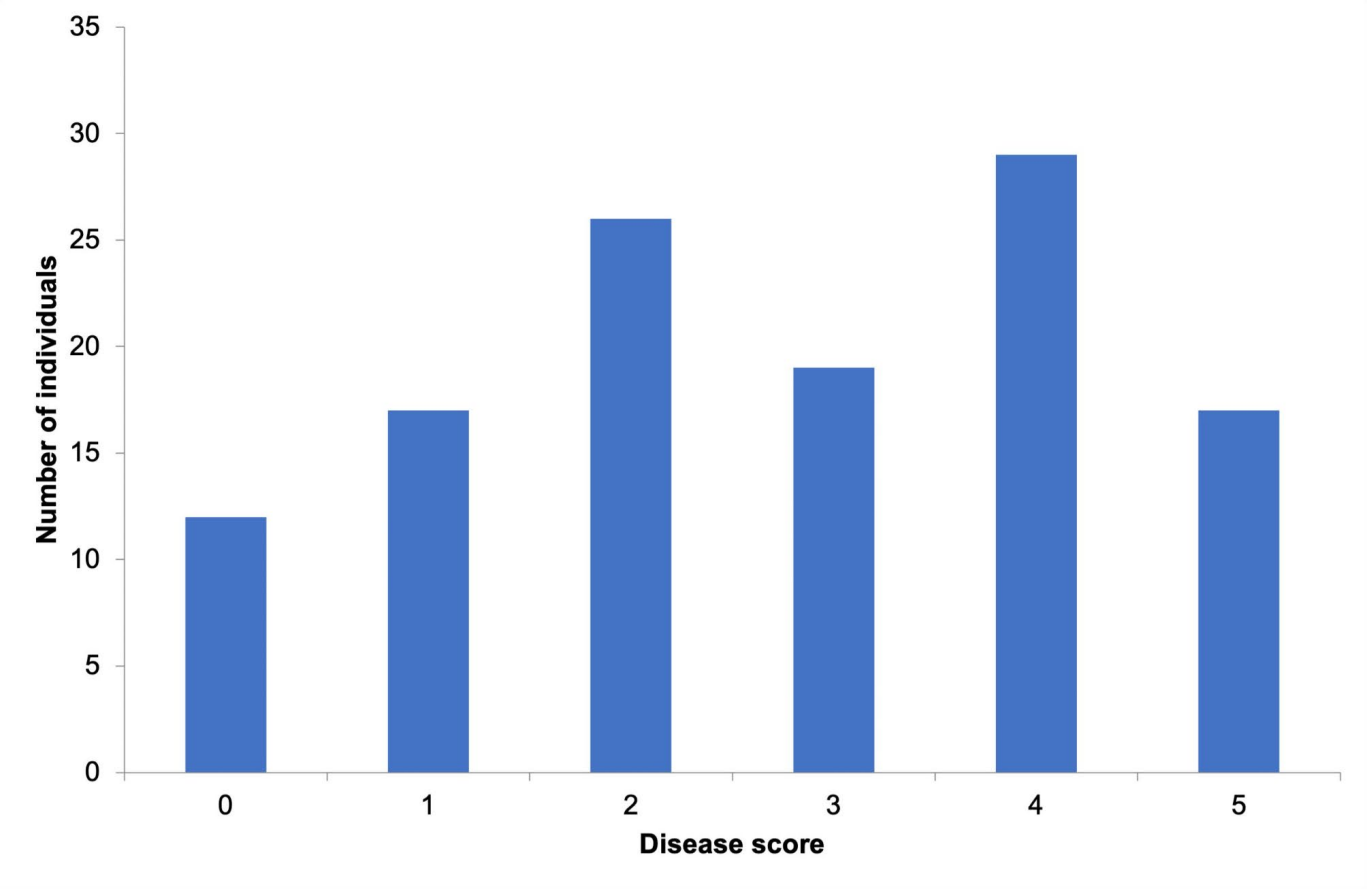
Disease score distribution for the 120 maize genotypes based on 0–5 scores.

The resistance to *Mp* for 120 different maize genotypes under controlled conditions was evaluated and disease symptoms were scored. The average disease score was calculated as 4.2. Out of 120 genotypes, 12 were classified as resistant, 43 as moderately resistant/tolerant, 48 as moderately susceptible/susceptible, and 17 as highly susceptible. As a result of the examination of the disease response distribution, it was determined that the population used exhibited a normal distribution suitable for the GWAS study. The normal distribution of genotypes is depicted in the histogram graph in Fig. 1. Analysis of variance of *Mp* disease scores reveals significant effects of maize genotypes on disease severity. The results of the analysis of variance are presented in Table 1. Analysis of variance showed that the resistance reactions to *Mp* among the 120 maize genotypes were statistically significant at *P* ≤ 0.05 level which shows that there are differences among the genotypes in terms of reaction to the pathogen infection. ANOVA assumptions, including homogeneity of variances and normality, were tested and confirmed to be accurate. In addition, the broad sense heritability score was calculated as 0.80.

**Table 1:** Analysis of variance results of disease severity and genotype.

	Sum of squares	df	Mean square	F	Sig.
Genotype	972.62	119	8.17	13.31	1.29
Error	147.33	240	0.61		
Total	1119.95	359			

*df*: degree of freedom; F: F-value; *Sig*. significance.

### Population structure analysis

**Fig. 2:**
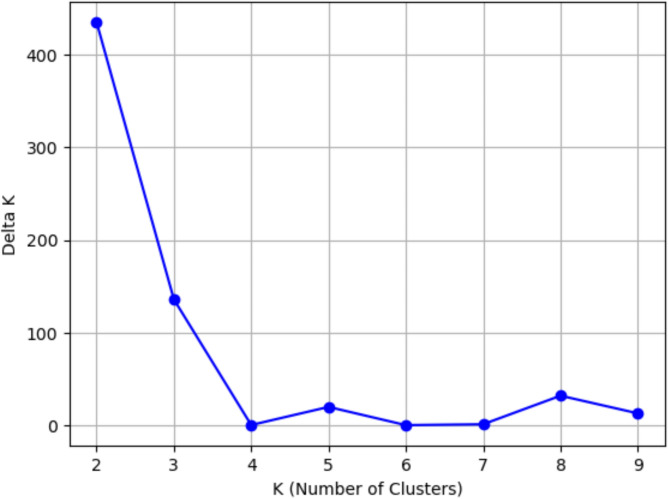
ΔK plot obtained from STRUCTURE HARVESTER software.

**Fig. 3:**
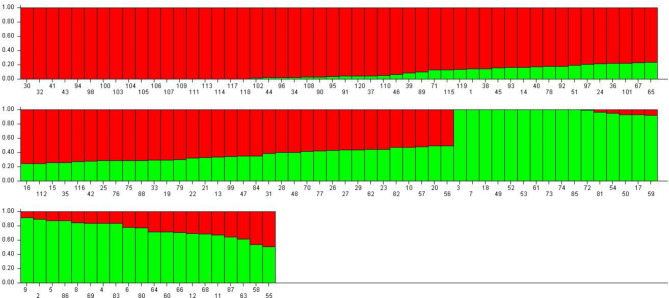
The population structure of the 120 maize genotypes was analyzed based on 37,470 DArT-seq markers using STRUCTURE.

A total of 79,166 SNPs were obtained through DArT analysis. The initial SNP data were subjected to strict filtration criteria allowing only SNPs with 95% call rate and 5% minor allele frequency (MAF) to remain, resulting in 37,470 high-quality SNPs. This process was performed to ensure the reliability of subsequent analyses. SNPs are uniformly distributed among chromosomes, with an average of 2,500 SNPs per chr. The highest SNP number was found in chr 1 with 4,129 SNPs and the lowest SNP number was found in chr 10 with 1,639 SNPs. Furthermore, the polymorphism information (PIC) content values of SNPs ranged from 0.01 to 0.5, with an average of 0.31. This indicates a moderate level of genetic diversity within the population. Following filtration, clean SNP data were utilized in STRUCTURE analyses with 50,000 iterations. The ΔK value was determined by conducting calculations for each group ranging from 1 to 10 using STRUCTURE HARVESTER software, with the peak observed at K = 2 (Fig. 2). The population was divided into two groups: population 1 (green) and population 2 (red), as shown in Fig. 3.

#### Association analysis and candidate genes

**Fig. 4:**
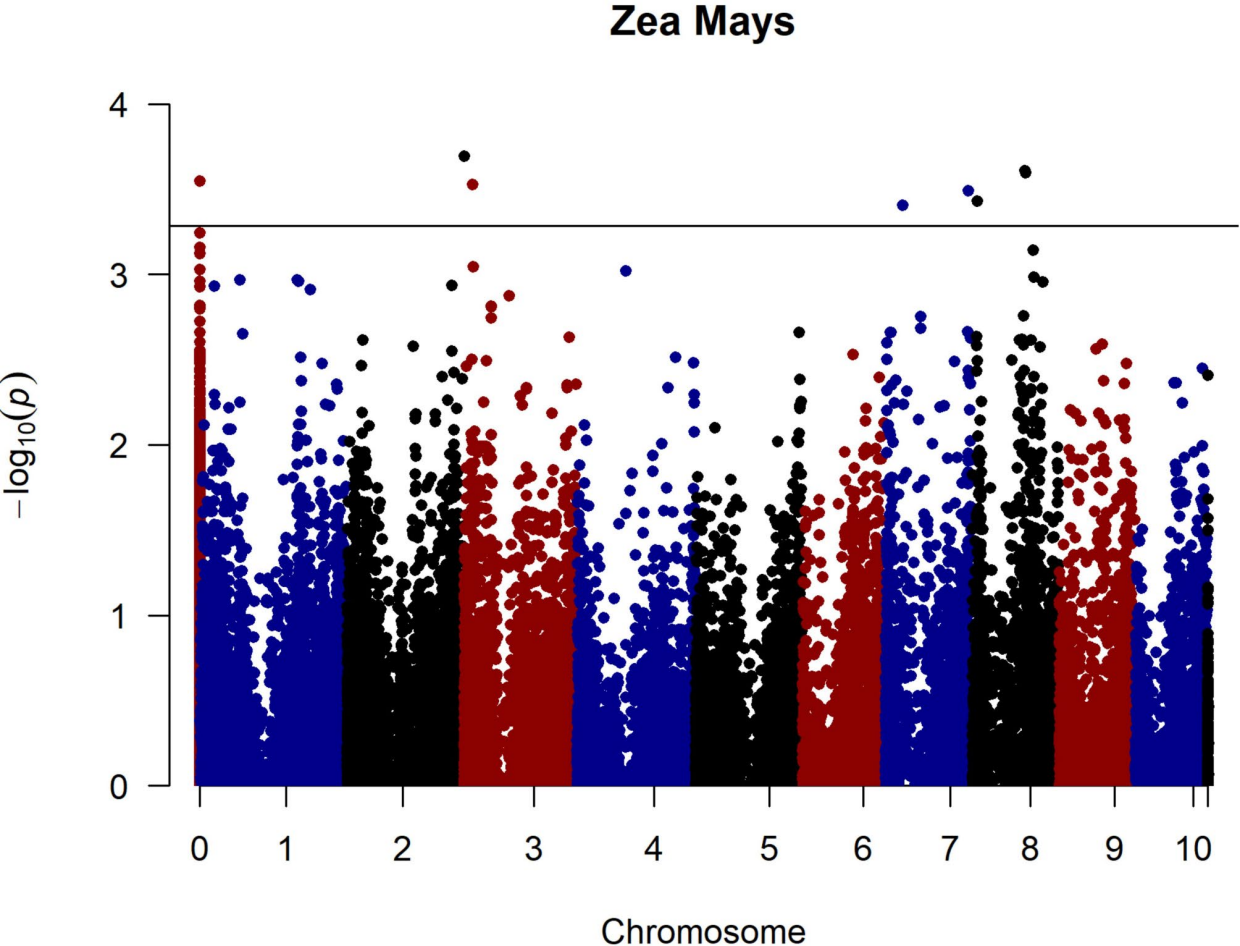
Manhattan plot of *Mp* resistance in 120 maize genotypes.

**Fig. 5:**
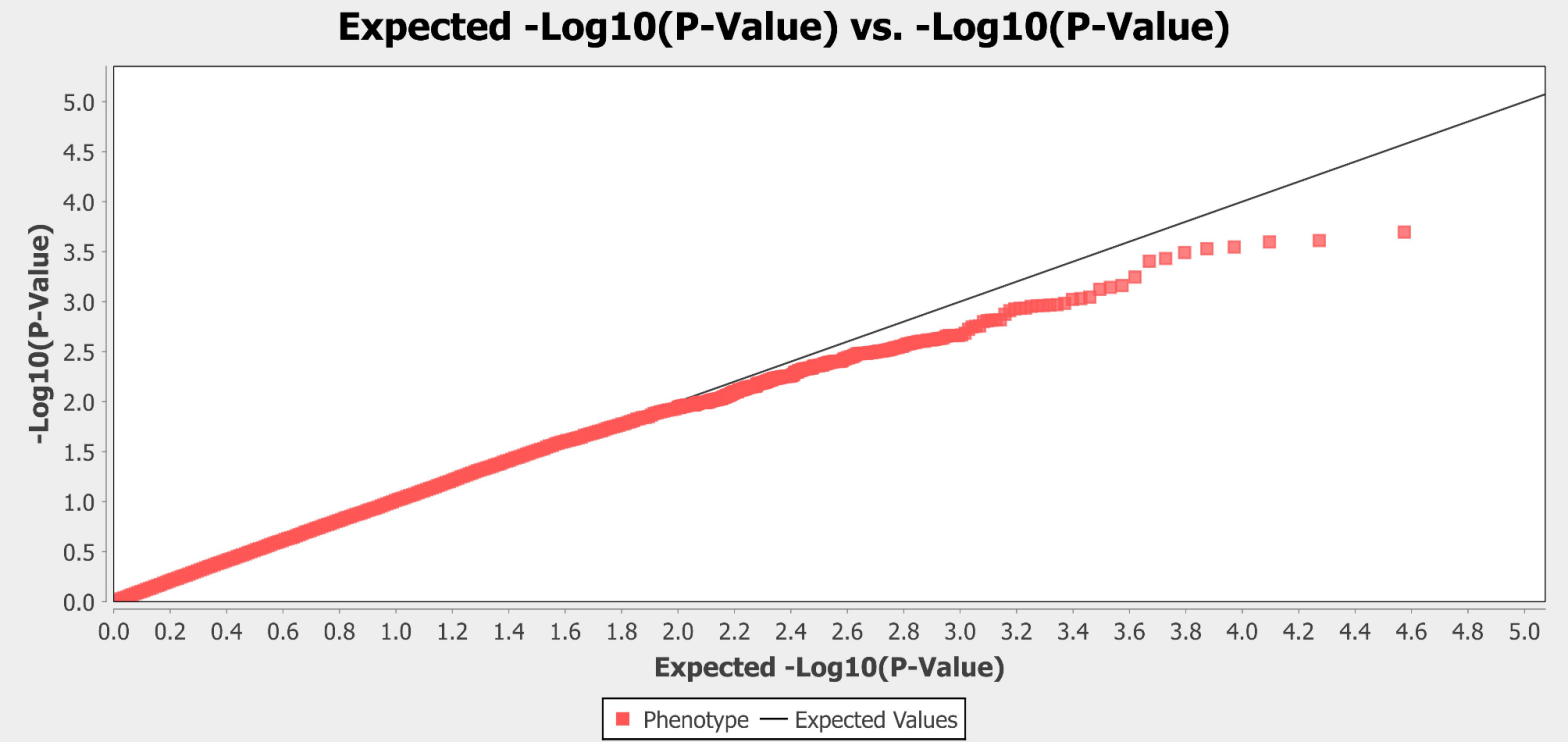
Quantile-quantile plot for the *Mp* resistance.

GWAS analysis was carried out in TASSEL software. From the option, the MLM (Q + K) model was chosen to analyze associations between marker and trait data. The MLM (Q + K) model decreases the false positive rate in analysis by accounting for both population structure and genetic linkages. This model adjusts the population structure using the Q matrix and genetic relationships through the K matrix, thereby yielding more precise results^21^. According to the GWAS analysis, seven SNPs were detected along the four different chromosomesexceeding the FDR corrections. FDR corrections (-log_10_P value is ≥ 3.28) were utilized to filter the most likely SNPs to avoid false positives. The Manhattan plot illustrates the distribution of SNPs across various chromosomes and identifies significant SNPs concentrated in specific regions (Fig. 4). After the filtering process, seven SNP markers associated with resistance were identified. The -log_10_P, R², and PIC values of the SNPs found were used to evaluate the significance and effect of these markers (Table 2). The -log_10_P value indicates the statistical significance of the SNP’s association with the phenotype, while the R² value reflects the proportion of phenotypic variation explained by this SNP. The PIC value expresses the SNP’s capacity to reflect genetic variation. SNP6999, located on chr 2, was found to be the most associated marker with the highest -log_10_P value (3.70). It was followed by SNP12080 and SNP34600 on chr 8 (-log_10_P, 3.61 and 3.60, respectively). As seen in the Q-Q plot, most data points are positioned on the diagonal line, indicating that the observed P values show a good fit to the expected distribution. This fit suggests that the P values were appropriately adjusted for multiple testing and that the analysis was robust to control for potential false positives (Fig. 5). Candidate genes were identified by screening up and down 100,000 base pairs of SNP locations, and 33 different genes with various activities were found. These genes are involved in disease resistance and play important roles in plant defence mechanisms such as cell wall synthesis, phytohormone signalling and activation of transcription factors. They also show the potential to alter cell wall composition, regulate stress responses and increase resistance to fungal pathogens. The candidate genes associated with the SNP markers and their functions are given in Supplementary Table S2.

**Table 2:** The list of SNP markers associated with *Mp* resistance.

Marker	Chromosome	Position	-log_10_*P*	*R* ^2^		PIC values
SNP6999	2	244,407,872	3.70	0.12		0.27
SNP12080	8	109,468,297	3.61	0.12		0.26
SNP34600	8	110,434,136	3.60	0.11		0.27
SNP22096	3	17,631,976	3.53	0.11		0.25
SNP892	7	173,715,255	3.49	0.11		0.32
SNP23394	8	9,998,386	3.43	0.11		0.24
SNP26238	7	36,306,357	3.40	0.11		0.49

## Discussion

*Mp* is one of the most destructive pathogens of maize production, causing major losses in maize yields. It causes yield loss not only in maize but also in soya bean^22,23^, canola^24^, sorghum^25,26^ and other plants^27–29^. For this reason, many studies have been carried out to determine the genetic factors that provide resistance in various plants. Statistical analysis of the disease resistance data obtained because of phenotypic observations revealed that there was significant diversity among the genotypes used in the study and that the data were normally distributed. However, environmental factors and growth conditions were reported to have a significant effect on resistance to fungal infections^30^. In this study, variation between genotypes was analyzed by ANOVA on phenotypic data and this variation was attributed to genetic factors. Broad sense heritability indicates the proportion of total phenotypic variation attributable to genetic factors and was found to be 0.80 in this study; therefore, it can be concluded that broad sense heritability demonstrates that the disease resistance trait is generally inherited genetically. This value was higher than those reported in the literature. The heritability value was determined as 0.54–0.67 in maize^31^ and approximately 0.6 in soybean^32^ in GWAS studies for resistance to *Mp*. These values indicated that resistance to *Mp* is highly heritable. Therefore, the high heritability of disease resistance is an indication of the reproducibility of the study and the reliability of its use in breeding studies.

In this study, the MLM (Q + K) model is preferred to correct the population structure. This model effectively eliminates potential false positives by taking into account random and fixed effects according to Henderson’s notation and provides precision in analysing complex traits^33^. Previous research has revealed that between the observed and expected -log_10_P values shown by the Q-Q plot, the G-test model exhibits the highest deviation, followed by the general linear model (GLM or G + Q model). However, the MLM model shows the least bias as it corrects for both population structure (Q) and relatedness (K)^31^. Furthermore, FDR (False Discovery Rate) correction was applied to reduce false positive associations. These methods are critical for improving the reliability of results in large-scale GWAS studies.

In the present study, a GWAS study was performed with a total of 37,470 SNPs by filtering SNPs obtained by the DArT-seq. As a result of the analyses, seven SNPs were identified with -log_10_P values ranging from 3.70 to 3.40, indicating the statistical significance of the effect of SNPs on phenotype. In addition, R^2^ values, which indicate how much of the phenotypic variance is explained by genotypes, were found to vary between 0.12 and 0.11. Considering the genetic basis of complex characteristics such as disease resistance, a single SNP is not expected to explain a large proportion of the phenotypic variance. Therefore, R² values around 0.1 can be predicted and the results obtained in this study are under the polygenic nature of complex traits. According to a study conducted in soybeans, 19 out of 35,683 clean SNPs obtained with Illumina Infinium SoySNP50K BeadChip were detected to be associated with the *Mp*^32^. In a different study, 3,780 clean SNP data in soybeans were also obtained using the same method. Among the SNPs obtained, six SNPs with R^2^ values ranging between 9.5 and 11.8% were reported to be associated with resistance^34^. These R^2^ values align with the findings of the current study, supporting the consistency and reliability of the results. However, only one GWAS study in maize has been identified so far. In the study in which 396 maize varieties adapted to Asia were used, filtering was performed according to call rate > 0.7 and MAF > 0.05, resulting in 296 K clean SNPs obtained by Genotyping by Sequencing (GBS) method^31^. In the present study, a more stringent call rate filter (> 0.95) was used to minimize false positives. A total of 19 SNP markers associated with disease resistance, with -log_10_P values ranging from 5.23 to 4.32, were identified. While the reported -log_10_P values in that study were statistically more significant than those observed here, lower heritability values (ranging from 0.67 to 0.54) were reported, likely due to the field conditions under which the study was conducted. Environmental factors beyond control may have negatively impacted the heritability estimates^30^. In contrast, the controlled conditions used in this study resulted in higher heritability values. Similar to the current study, most SNPs were identified on chromosomes 3, 6, and 8^31^. Additionally, for the first time, genomic regions associated with *Mp* resistance on chromosome 7 in maize were identified in this study.

Gene loci likely to be associated with disease resistance were identified through association mapping. However, candidate genes can only be predicted by association mapping studies, and further research is needed to determine the role of these genes in the resistance mechanism^35^. SNP6999, located on chr 2, is localized between several gene regions such as protein kinases, transcription factors, and ATP-DNA binders. The defense mechanisms of plants against pathogens are quite diverse. Among these, phytohormones such as salicylic acid and ethylene play an important role. The gene LOC100502174 has not yet been characterized in the maize genome, but the protein it expresses has an AP2/ERF domain. Ethylene-containing elements, also known as Ethylene-Responsive Elements (ERE), induce the expression of some plant defense-related genes^36^. ERE binding factors (ERF) are transcription factors that recognize ERE sites. In previous studies, the association of ERF sites with resistance to *Mp* has been demonstrated^37–39^. Moreover, the most basic defense mechanism of plants is the cell wall. Cell walls are composed of cellulose, hemicellulose, and pectins. The LOC103648302 gene is involved in the synthesis of xyloglucan (hemicellulose), an important component of the cell wall. In this regard, it may be related to disease resistance.

Only a small number of genes are physically close to SNP 22,096. The gene LOC100272653 is the most important of these. This region affects the morphology and physiology of the cell wall and contains domains of pectin methyltransferase. Cell respiration and photosynthetic efficiency can be changed by pectin methyltransferase. It can also change the plasticity of cell walls^40,41^. This gene is likely to contribute to fungal pathogen resistance by modifying the cell wall^40^.

SNP 892 and SNP 26,238 were found to be close to nine different gene regions, including various protein kinases, transmembrane proteins, and transcription factors. Prior research has indicated that ubiquitinated proteins^42^ and protein kinases^39^ are involved in host defense. Moreover, pectin methylesterase activity was detected in LOC100274042, which hardens and consolidates the cell wall against various pathogens and generates damage signals to activate defense mechanisms^43^.

The chr 8 was found to have a significant association with resistance to *Mp*. This region included three different SNPs that indicate ten different gene regions. SNP 12,080 was found to be close to the *mbd*101 gene, which expresses zinc-finger regions with proven activity in plant defense^44,45^, and to the cytochrome P450 gene^46^, which is involved in secondary metabolite synthesis and oxidation and reduction reactions. SNP 23,394 was located generally close to genomic regions containing various kinases such as serine/threonine, cysteine, and rust resistance. All these kinases are associated with polysaccharides in the cell wall and thus contribute to the cell wall defense mechanism^47^.

## Conclusion

The genetic background of *Mp* resistance in maize was investigated by GWAS. In this study, SNP markers were generated using DArT-seq technology for the first time among the association studies for *Mp*. In GWAS analyses, seven SNPs were found to be significantly associated. Several candidate gene regions were identified on chromosomes 2, 3, 7, and 8 that contribute to disease resistance in different ways. The SNP markers associated with resistance to *Mp* identified in this study could be used in marker-assisted selection (MAS), including MAS backcrossing and MAS for quantitative traits, to select lines resistant to *Mp* disease in future maize breeding programs. Moreover, 12 maize genotypes found to be resistant to the disease have the potential could be used as donor parents in breeding programs.

## Materials and methods

### Plant material and DNA isolation

The plant materials were kindly received from Polen Seed Co. in Türkiye. A list of 120 genotypes that were used listed in Supplementary Table S1. Liquid nitrogen was used to freeze fresh leaves that were obtained from the genotypes and weighed 0.1 g. After that, they were grounded to a fine powder using a tissue lyser (Technogene, Türkiye). The Qiagene DNA isolation kit (Catalogue No./ID: 69181) was applied to extract DNA from the powdered leaves. The quality of the extracted DNA was examined on an agarose gel, and the quantity was measured using a Nanodrop One (Thermo Sci. Co.). The isolates of the *Mp* were collected from previous studies and pathogenicity was determined, and then the most aggressive pathogen was used for the determination of genotype reaction to *Mp* resistance. Following a 7-day incubation period of the fungus in potato dextrose agar medium, ten agar disks were added to each bottle containing oat medium. The bottles were then incubated at 25 ± 2 °C for 15–20 days. After the completion of incubation, a mixture of composted animal manure, sterilized (45 min at 121 °C) soil, and fine sand (2:1:1) was combined with 100 g of inoculum per 1 kg of soil and the mixture was then incubated for five days.

### Inoculum preparation and phenotypic analysis

For the genotype reaction studies, the fungal inoculum was first developed by filling 500 ml glass bottles with a mixture of sand-water-cornmeal (9:2:1) or bran. Bottles with media were sterilized in an autoclave at 121 °C for 1 h on 2 consecutive days. Fungal isolates and sclerotia, developed in PDA, were placed in each bottle in the form of 10 agar discs, 5 mm in diameter, and incubated at 30 ± 2 °C for 15–20 days. Once the fungal incubation was completed, a mixture of sterilized garden soil (121 °C for 45 min), peat, perlite, and fine sand (2:1:1) was filled into trays. The inoculum, prepared in the bottles, was then mixed into the soil at a rate of 100 g inoculum per 1 kg of soil and incubated for 4–5 days. After the sterilization with 2% sodium hypochlorite 6 seeds of each genotype were sown in 21 cm diameter pots filled with a mixture of sterile soil, fine sand, peat (2:1:1) containing inoculum. The cultivar P31G98 was utilized as a positive control due to its being known to be sensitive to *Mp*^48^. Negative control plants were planted in disease-free pots.

**Fig. 6:**
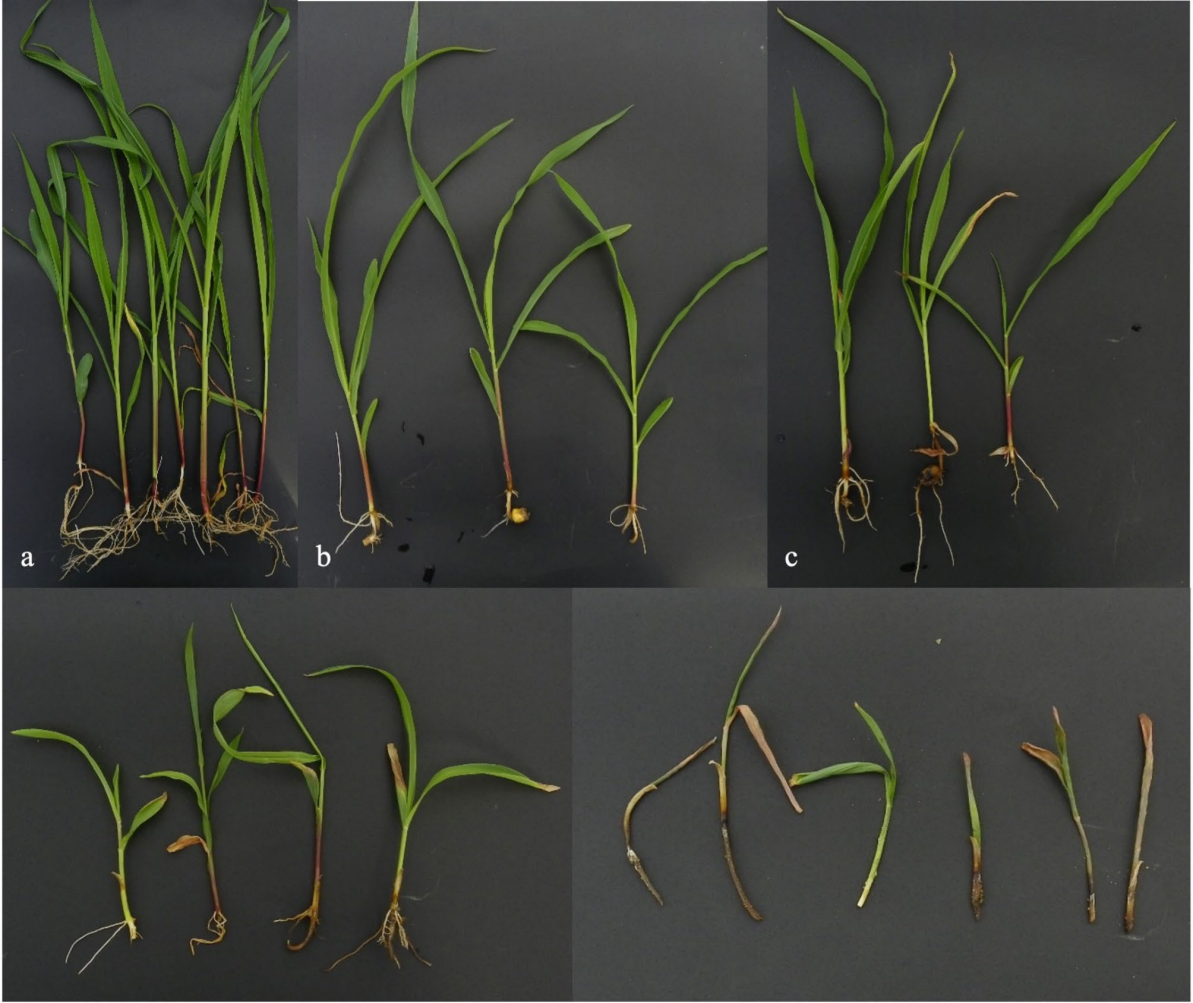
Plants representing the 0–5 scale used for scoring corn genotypes. 0: No disease symptoms observed in the plant (**a**); 1: General growth retardation in the plant (**b**); 2: Growth retardation in the plant, chlorosis in the leaves, and mild root rot (**c**); 3: Growth retardation in the plant, severe root rot, wilting, and intense chlorosis (**d**); 4: Significant growth retardation, complete root rot in the plant (**e**); 5: No germination observed.

The pots were maintained in climate chambers at 30 °C, 30–45% humidity, and a 16-hour light/8-hour dark cycle. Plants were watered as necessary, ensuring that the soil moisture was not too high^49^. The pathogenicity trials were set up in a randomized complete block design with 3 replicates. Each pot was considered one replicate. The disease severity in the plants was be assessed using a 0–5 scale^48^ (Fig. 6). After 40 days under the controlled conditions, 40-day-old plants were uprooted, disease symptoms were scored and the disease severity index (DSI) on roots and stems was calculated using the Townsend-Heuberger formula (S = score, P = number of plants, TP = total number of plants, HS = highest score) (Table 3)^50^.

**Table 3:** Criteria used in phenotyping and corresponding values^50^.

Scale	Description	Phenotype	DSI
0	No disease symptoms observed in the plant	Resistant	0–20
1	General growth retardation in the plant	Moderately resistant	20–40
2	Growth retardation in the plant, chlorosis in the leaves, and mild root rot	Tolerant	40–60
3	Growth retardation in the plant, severe root rot, wilting, and intense chlorosis	Moderately susceptible	60–80
4	Significant growth retardation, complete root rot in the plant	Susceptible	80–99
5	No germination observed	Highly susceptible	100

$$Disease\;Severity\;\%=\sum \frac{S\times P}{TP\times HS}\times 100$$

Genotype reactions were defined according to DSI and phenotypes were arranged in 6 groups (Table 3).

### Statistical analysis of phenotypic data

Variance analyses (ANOVA) were conducted using the SPSS software package (version 12.0 J; SPSS Inc., Chicago, IL, USA). At the *p*≤ 0.05 significance level, the variances according to the disease severity and genotype interactions were identified. Broad sense heritability score calculated as described by^51^.

### Molecular marker analysis

SNP markers were determined using DArT analysis (Diversity Arrays Technology Pty. Ltd., Canberra, Australia). Extracted DNA was cut with *Pst* I-*Mse* I enzymes and ligated to adapters compatible with these enzymes as described previously^52^. PCR and adapter ligation were performed following established protocols^53^. Sequencing was conducted using Illumina HiSeq 2000 (Illumina Inc., USA).

### Population structure and genome-wide association mapping

The raw SNP data obtained from sequencing were filtered based on a 95% call rate and a minor allele frequency (MAF) greater than 5%. Subsequently, the population structure was determined using STRUCTURE software to identify groups within the population that exhibited discriminant allele frequencies. The number of groups in the population is indicated by the K value, which is defined as the allele frequencies at each locus. For every group, a range of 1 to 10 was calculated to find the most favorable K value. Ten replicates were analyzed for every K value, and 50,000 Markov Chain Monte Carlo (MCMC) replicates were performed for the burn-in length and burn-in period. The STRUCTURE HARVESTER^54^ software was utilized to determine the ΔK value with the highest probability for every K value that was obtained.

The association between SNP markers and resistance of maize genotypes to *Mp* was determined in the TASSEL software^55^. For this purpose, the significance levels of the relationship between phenotypic data, marker data, and Q matrix, which represents the population structure, were analyzed with TASSEL software. The Mixed Linear Model (MLM) model was employed in this study to minimize false positives by integrating population structure and genetic relationships. It utilizes the Q matrix for population adjustments and the K matrix for genetic linkage, resulting in enhanced accuracy in the analysis^56^. FDR (False Discovery Rate) is a method used to control the proportion of false positive results in statistical testing^57^. The FDR correction threshold was calculated to minimize the likelihood of detecting false positive SNPs^58^. This approach enables a more accurate identification of significant SNPs while maintaining the integrity of the analysis. When FDR Correction was exceeded, SNP markers were considered statistically significant. The visual representation of these markers exceeding the FDR threshold line was drawn using Manhattan blocks. In order to visualize the relationship between observed and expected phenotypic data and P values, quantile-quantile (Q-Q) plots were also drawn^56^.

### Candidate genes

SNP markers linked to *Mp* in maize were examined using Sequence Viewer 3.49.0 on the National Center for Biotechnology (NCBI) database to identify potential genes. Analysis of candidate genes was carried out 100 K upstream and 100 K downstream of the marker’s positions.

## Electronic supplementary material

Below is the link to the electronic supplementary material.

Supplementary Material 1

## Data Availability

The data generated from the study will be provided by the corresponding author upon request.
