# Supplementary material for: Genome-wide association study for resistance to Macrophomina phaseolina in maize (Zea mays L.)
Source: Sci Rep. 2025 Mar 6;15:7794. doi: 10.1038/s41598-025-87798-8 (PMC11882914; doi:10.1038/s41598-025-87798-8)
Supplement: Supplementary file 1 — Supplementary Material 1 [file 41598_2025_87798_MOESM1_ESM.docx]

**Genome-wide association study for resistance to *Macrophomina phaseolina* in maize (*Zea mays* L.)**

Gizem Oder^1^, Semiha Yuceer^2^, Canan Can^3^, Muhammed Bahattin Tanyolac^1^, Duygu Ates^1*^

*^1^Department of Bioengineering, Ege University, Izmir, Turkey*

*^2^Phytopathology Department, Biological Control Research Institute, Adana, Turkey*

*^3^Department of Biology, Gaziantep University, Gaziantep, Turkey*

**Table S1.** The list of maize genotypes used in the study and disease scores

| Genotype Number | Genotype Name | Disease Score | Genotype Number | Genotype Name | Disease Score |
| --- | --- | --- | --- | --- | --- |
| 1 | PLF4001 | 0 | 61 | PLF7024 | 4 |
| 2 | PLF5003 | 0 | 62 | PLF7025 | 4 |
| 3 | PLF6001 | 0 | 63 | PLM7026 | 5 |
| 4 | PLF6012 | 1 | 64 | PLF7028 | 5 |
| 5 | PLF6014 | 4 | 65 | PLF7029 | 5 |
| 6 | PLF6017 | 0 | 66 | PLF7030 | 5 |
| 7 | PLF6019 | 0 | 67 | PLF7033 | 5 |
| 8 | PLF6020 | 0 | 68 | PLF7035 | 3 |
| 9 | PLF6022 | 0 | 69 | PLF7039 | 2 |
| 10 | PLF6023 | 1 | 70 | PLF7040 | 1 |
| 11 | PLF6028 | 1 | 71 | PLF7044 | 2 |
| 12 | PLF6029 | 4 | 72 | PLF7045 | 2 |
| 13 | PLF6030 | 3 | 73 | PLF7051 | 2 |
| 14 | PLF6032 | 3 | 74 | PLF7052 | 2 |
| 15 | PLF6035 | 4 | 75 | PLF7056 | 1 |
| 16 | PLF6036 | 4 | 76 | PLF7058 | 0 |
| 17 | PLF6038 | 0 | 77 | PLF7059 | 1 |
| 18 | PLF6039 | 0 | 78 | PLF7060 | 2 |
| 19 | PLF6041 | 2 | 79 | PLF7061 | 1 |
| 20 | PLF6043 | 3 | 80 | PLF7065 | 2 |
| 21 | PLF6044 | 2 | 81 | PLF7066 | 1 |
| 22 | PLF6046 | 1 | 82 | PLF7067 | 2 |
| 23 | PLF6048 | 3 | 83 | PLF7068 | 4 |
| 24 | PLF6050 | 1 | 84 | PLF7070 | 2 |
| 25 | PLF6052 | 4 | 85 | PLF7074 | 2 |
| 26 | PLF6058 | 3 | 86 | PLF8004 | 3 |
| 27 | PLF6059 | 4 | 87 | PLF8005 | 1 |
| 28 | PLF6060 | 2 | 88 | PLM3001 | 2 |
| 29 | PLF6061 | 1 | 89 | PLM4002 | 2 |
| 30 | PLM7016 | 5 | 90 | PLM4003 | 1 |
| 31 | PLF6064 | 1 | 91 | PLM5002 | 2 |
| 32 | PLM7025 | 4 | 92 | PLM5003 | 2 |
| 33 | PLF6066 | 2 | 93 | PLM5004 | 2 |
| 34 | PLM7005 | 4 | 94 | PLM5005 | 3 |
| 35 | PLF6069 | 4 | 95 | PLM5006 | 1 |
| 36 | PLM7012 | 3 | 96 | PLM6005 | 5 |
| 37 | PLM7014 | 3 | 97 | PLM7028 | 5 |
| 38 | PLF6073 | 4 | 98 | PLM6009 | 2 |
| 39 | PLM7024 | 5 | 99 | PLM6014 | 4 |
| 40 | PLM7015 | 4 | 100 | PLM6019 | 3 |
| 41 | PLM7006 | 4 | 101 | PLM6022 | 2 |
| 42 | PLM7036 | 3 | 102 | PLM6023 | 3 |
| 43 | PLM7038 | 3 | 103 | PLM6026 | 4 |
| 44 | PLM8001 | 3 | 104 | PLM6030 | 2 |
| 45 | PLM8013 | 2 | 105 | PLM6031 | 3 |
| 46 | PLM8014 | 3 | 106 | PLM6032 | 2 |
| 47 | PLF6087 | 4 | 107 | PLM6035 | 2 |
| 48 | PLF6088 | 5 | 108 | PLM6036 | 3 |
| 49 | PLF6090 | 4 | 109 | PLM7008 | 4 |
| 50 | PLF6091 | 4 | 110 | PLM6038 | 4 |
| 51 | PLF6086 | 5 | 111 | PLM6039 | 4 |
| 52 | PLF7005 | 5 | 112 | PLM6041 | 0 |
| 53 | PLF7012 | 5 | 113 | PLM6042 | 0 |
| 54 | PLF7013 | 5 | 114 | PLM6043 | 1 |
| 55 | PLF7015 | 5 | 115 | PLM6044 | 3 |
| 56 | PLF7017 | 4 | 116 | PLM6045 | 4 |
| 57 | PLF7018 | 5 | 117 | PLM6047 | 4 |
| 58 | PLF7019 | 5 | 118 | PLM6048 | 4 |
| 59 | PLF7020 | 4 | 119 | PLM7021 | 2 |
| 60 | PLF7021 | 4 | 120 | PLM7033 | 1 |

**Table S2** The list of candidate genes within 100 K downstream and 100 K upstream of SNPs

| **SNP ID** | **Chromosome** | **LOC ID** | **Gene Name** | **GO Terms Function** |
| --- | --- | --- | --- | --- |
| SNP 6999 | *Chr2* | LOC100502174 | Uncharacterized protein | DNA-binding transcription factor activity |
|  |  | LOC118476451 | Vicilin-like seed storage protein At2g18540 | Undefined function |
|  |  | LOC100192067 | Ribonucleoprotein like protein | RNA Binding |
|  |  | LOC541963 | Chromatin complex subunit A101 | ATP binding, ATP-dependent chromatin remodeler activity, DNA binding |
|  |  | LOC103648304 | Uncharacterized protein | Undefined function |
|  |  | LOC101027138 | TPR domain-containing protein | Histone binding |
|  |  | LOC103648302 | Probable fucosyltransferase 8 | Galactoside 2-alpha-L-fucosyltransferase activity (involved in cell wall biogenesis/xyloglucan biosynthetic process) |
|  |  | LOC100281440 | Uncharacterized protein | Protein kinase activity and ATP binding |
|  |  | LOC100280324 | Succinate dehydrogenase4 | Electron transfer activity, flavin adenine dinucleotide binding, and succinate dehydrogenase (quinone) activity |
|  |  | LOC103648298 | Uncharacterized protein | Zinc ion binding |
|  |  | LOC103649501 | 14-3-3-like protein GF14-D | Involved in signal transduction |
| SNP 22096 | *Chr3* | LOC100272653 | Uncharacterized protein | Methyltransferase activity and pectin metabolic process |
|  |  | LOC100193742 | Uncharacterized protein | Involved in transmembrane transport |
|  |  | LOC103649787 | Uncharacterized protein | Oxidative phosphorylation uncoupler activity |
| SNP 892 | *Chr7* | LOC100193267 | Uncharacterized protein | Protein K11-linked ubiquitination |
|  |  | LOC100193344 | Uncharacterized protein | GTP binding and GTPase activity |
|  |  | LOC100272748 | SNARE-associated Golgi protein family | Undefined function |
|  |  | LOC103634409 | BTB/POZ and MATH domain-containing protein 1 | Protein ubiquitination |
|  |  | LOC103633471 | Transcription factor PCF8 | DNA-binding transcription factor activity (involved in regulation of secondary shoot formation) |
|  |  | LOC100274042 | Uncharacterized protein | Pectin acetylesterase activity |
|  |  | LOC100193458 | p21-activated protein kinase-interacting protein 1-like | Protein binding |
| SNP 26238 | *Chr7* | pht11 | Phosphate transporter protein11 | Phosphate transmembrane transporter activity |
|  |  | LOC103632249 | Adenylate kinase isoenzyme 6 homolog | ATP binding, ATP hydrolysis activity, and adenylate kinase activity |
| SNP 12080 | *Chr8* | mbd101 | Methyl-binding domain 101 | DNA-binding and zinc ion binding |
|  |  | LOC109941845 | Uncharacterized protein | Undefined function |
|  |  | LOC103635678 | Pyruvate, phosphate dikinase 2-like | ATP binding, protein kinase activity, and pyruvate, phosphate dikinase activity |
|  |  | LOC109941639 | Cytochrome P450 734A1 | Heme binding, iron ion binding, monoxygenase activity, and oxidoreductase activity (acting on paired donors, with incorporation or reduction of molecular oxygen) |
| SNP 34600 | *Chr8* | LOC100283286 | mTERF family protein | Double-stranded DNA binding |
|  |  | LOC100279213 | Arm Repeat Proteın Interactıng Wıth Abf2 | Protein binding |
| SNP 23394 | *Chr8* | LOC103634767 | Cysteine-rich receptor-like protein kinase 37 | ATP and polysaccharide binding, protein kinase activity |
|  |  | LOC100193171 | Putative protein kinase superfamily protein | ATP and polysaccharide binding, protein kinase activity |
|  |  | LOC542671 | Ser/Thr receptor-like kinase 1 | ATP and polysaccharide binding, protein kinase activity |
|  |  | LOC103636743 | Rust resistance kinase Lr10 | ATP binding, protein kinase activity (involved in protein phosphorylation) |
